# Effects of Electroacupuncture on PGC-1**α** Expression in Brown Adipose Tissue

**DOI:** 10.1155/2013/625104

**Published:** 2013-12-30

**Authors:** Hongyin Du, Cuisong Zhou, Hui Wu, Tianyue Shan, Zhenjue Wu, Bin Xu, Yubin Zhang

**Affiliations:** ^1^State Key Laboratory of Natural Medicines, Department of Biochemistry, China Pharmaceutical University, Nanjing 210009, China; ^2^Institute of Chinese Materia Medica, Shanghai University of Traditional Chinese Medicine, Shanghai 201203, China; ^3^Nanjing University of Chinese Medicine, Nanjing 210023, China

## Abstract

The inducible coactivator PGC-1**α** plays master regulator in mitochondrial biogenesis and thermogenesis in brown adipose tissues (BATs). BAT is a natural antiobesity organ which dissipates chemical energy in the form of heat through specialized mitochondrial protein UCP-1. Eletroacupuncture (EA) has been widely used as an alternative treatment for obesity and its related disorders such as type 2 diabetes. The molecular mechanism of electroacupuncture on treatment of obesity is still unclear. We hypothesized that electroacupuncture induced PGC-1**α** expression to increase the energy expenditure in BAT. Rats were randomly divided into control group and electroacupuncture treatment group. We investigated the effects of electroacupuncture at Zusanli (ST36) acupoint on the expressions of PGC-1**α** and its associated genes in the BAT of rats using real-time PCR and western blotting. We found that electroacupuncture effectively induces the expression of PGC-1**α** and UCP-1 by 4-fold and 5-fold in the BAT of rats, respectively. Our results indicated that the molecular mechanism of electroacupuncture for the treatment of obesity may be, or at least partially, through induction of both PGC-1**α** and UCP-1 expressions to increase energy expenditure in BAT.

## 1. Introduction

It is well known that obesity develops when energy intake exceeds energy expenditure. Sedentary lifestyle and ready supply of calorie-dense food have caused a massive rise in the prevalence of obesity. Obesity has reached epidemic proportions. Obesity favors the development of insulin resistance, type 2 diabetes mellitus, and cardiovascular disease. These complications related to obesity contribute substantially to health care costs and increasing mortality rate [1, 2]. Current conventional therapeutic strategies for obesity cannot achieve adequate weight control in patients; complementary types of treatment are also performed [3].

Acupuncture, one of the oldest healing practices, represents the most rapidly growing complementary therapy which is recognized by both the National Institutes of Health (NIH) and World Health Organization (WHO) [4, 5]. Acupuncture and electroacupuncture at Zusanli (ST36) acupoint have been observed to reduce obese body weight and improve obesity-related insulin resistance [6]. Acupoint Zusanli (ST36) is the most commonly used and effective acupoint for the treatment of obesity in experimental research and clinical studies. However, the underlying molecular mechanisms are still unclear [6]. Two types of adipose tissue have been found in mammals, white adipose tissue (WAT) and brown adipose tissue (BAT). WAT is responsible for TG storage and BAT is a thermogenic tissue whose main function is to produce heat. Recent studies show that adult humans retain metabolically active BAT depots which can be induced in response to cold and sympathetic nervous system (SNS) activation, suggesting BAT might be a potential pharmacological and genetic target to treat human obesity [7–10]. When BAT is activated, it requires the uptake of free fatty acids mostly from white adipose tissue (WAT). The oxidation of free fatty acids in the mitochondria of BAT releases heat by the uncoupling protein 1 (UCP-1) [11, 12]. Brown adipose tissue (BAT) represents a natural target for antiobesity [13].

PGC-1*α* was first identified as a coactivator of transcriptional factor PPAR*γ* and it is highly expressed in BAT upon exposure of mice to cold [14]. PGC-1*α* plays a central role not only in adaptive thermogenesis through the upregulation of UCP-1 in BAT, but also in gluconeogenesis in liver and fiber type switching in muscle [15, 16]. In each of these cellular contexts, PGC-1*α* is regulated by signaling inputs that increase the transcription of the PGC-1*α* gene and activity of PGC-1*α* protein [17]. In this study, we hypothesized that electroacupuncture (EA) reduces body weight of obese rats through the induction of PGC-1*α* expression in BAT. To test our hypothesis, we investigated the effect of electroacupuncture at ST36 acupoint on the expression of PGC-1*α* in the BAT of rats using molecular biology approaches. The results indicated that the expression of PGC-1*α* in the BAT of rats is induced by electroacupuncture at acupoint Zusanli (ST36).

## 2. Materials and Methods

### 2.1. Experimental Animals

Six-week-old SD male (200–220 g) rats were obtained from Shanghai Laboratory Animal (CoSLAC.), Ltd., China. Rats were acclimated for 1 week in a light-controlled room (12 : 12 h light-dark cycle) under constant temperature (22-23°C). Standard rat chow diet and water were available *ad libitum*. Obese rats were obtained in our laboratory by feeding high-fat diet (56% of calories from fat, caloric density 4.73 kcal/g) for 6 weeks. The body weight of obese rats was 20% more than normal rats. All experiments were approved by the Institutional Animal Care and Use Committee at China Pharmaceutical University and adhere to the Jiangsu Provincial Guidelines for the use of experimental animals.

### 2.2. Electroacupuncture Protocols

**Figure 1:**
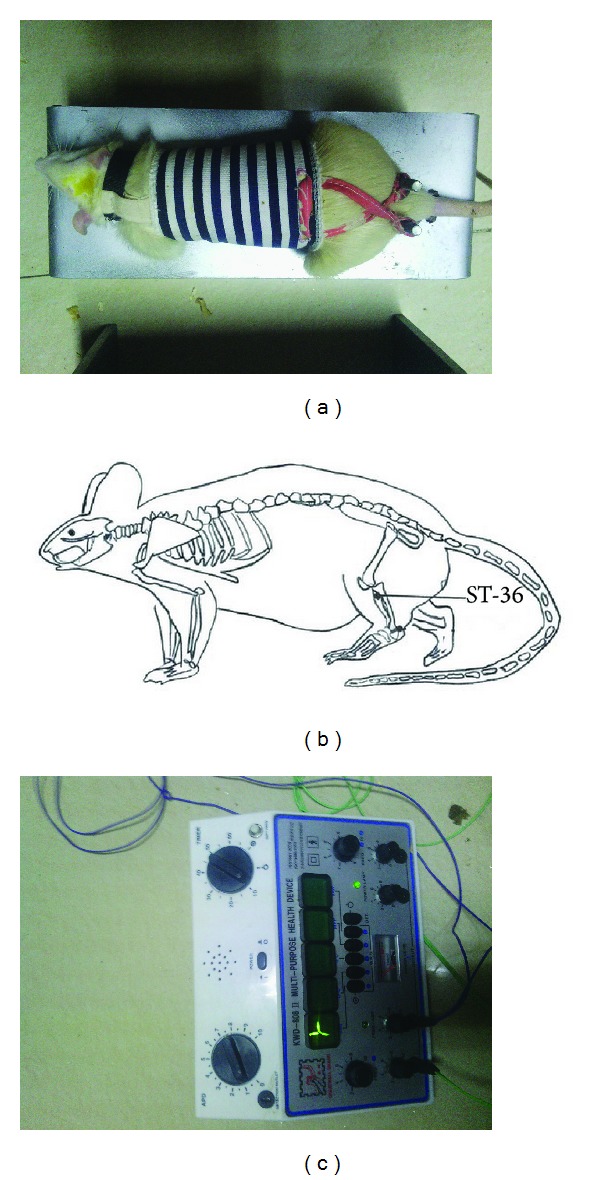
Electroacupuncture instrument and diagram of acupoint Zusanli (ST36). (a) Homemade holder for fixation of rat under conscious and comfortable states without anesthesia treatment. (b) Schematic diagram of Zusanli (ST36) located in the hind leg of rats and corresponding to the equivalent acupoints in humans. (c) Electrostimulator specific for acupuncture with adjustment of electroacupuncture parameters, such as electric frequency and electric current.

Rats were fixed on homemade holder instrument (Figure 1(a)) for 10 min each time and twice a day for 5 days without acupuncture operation only to train rats to acclimate the acupuncture manipulation. After one week of acclimatization, rats were randomly divided into control group and EA treatment group (6 rats per group). Rats in the electroacupuncture group were inserted an acupuncture needle (40 × 0.4 mm, Acuneedle Co., China) at Zusanli (ST36) acupoint (Figure 1(b)) into 5 mm without anesthesia; the acuneedle was turned around gently and shortly by hand for 5 times. Then, the acuneedle was linked to an electrostimulator (Figure 1(c)) (G6805-2A, Shanghai Huayi Medical Instrument Factory, China) and given electrical stimulation (8 Hz, 2 mA) for 10 minutes each time, twice (10 AM and 4 PM) every day. Rats in the control group were fixed as EA group but were not given electrical stimulation. In all experiments, rats were killed by decapitation 4 hours after last acupuncture; then, BAT tissues were rapidly removed from rats, frozen immediately in liquid nitrogen, and kept at −80°C until use.

### 2.3. RNA Isolation and Real-Time PCR (RT-PCR) Analysis

Total RNA was extracted from BAT tissues using TRIzol reagent (Invitrogen, USA). According to the manufacturer's protocol, 50–100 mg of BAT tissues was mixed with 1 mL of TRIzol and homogenized using Homogenizer (IKA-T-10, Germany). The tissues were mixed with 1 mL of TRIzol. RNA was separated from protein and DNA by the addition of chloroform and precipitated in 2 volumes of cold pure ethanol. After a 75% ethanol wash and resuspension in 20 *μ*L of DEPC-treated ddH_2_O, RNA samples were quantified by spectrophotometry. 1 *μ*g of total RNA was reverse-transcribed using oligo-(dT)_18_ primers and M-MLV reverse transcriptase (Roche, Germany) according to the protocol of First Strand cDNA Synthesis Kit (Roche, Germany) and 50 ng of cDNA was used as template for quantitative RT-PCR on Step One Plus System (Applied Biosystems, USA). PCRs were conducted using the primers (Table 1) which aresimilar to our previous study [18]. RT-PCR reactions were carried out in a 20 *μ*L volume containing 1XFast Start Universal SYBR Green Master (ROX) (Roche, Germany), 50 ng cDNA, and 0.3 *μ*M forward and reverse primers (each). Thermal cycling conditions were 95°C for 10 min and then 40 cycles of 95°C for 15 s and 57°C for 30 s. Target gene expression in each sample was normalized to the endogenous control gene cyclophilin. The relative expression among the different conditions was determined using the ΔΔ*C*_*T*_ method as outlined in the Applied Biosystems protocol for RT-PCR.

### 2.4. Western Blot Analysis

Rat brown adipocytes tissues were homogenized and sonicated in ice-cold lysis buffer (RIPA) containing 50 mM Tris/HCl (pH 7.4), 150 mM NaCl, 1% Nonidet P40, 0.25% sodium deoxycholate, 1 mM EDTA, and protease inhibitor cocktail (Roche, Switzerland). Cell lysates were centrifuged at 10000 ×g, supernatant was collected and protein concentration in supernatant was determined using the bicinchoninic acid (BCA) method. Equal amounts of protein (100 *μ*g) were loaded on 10% polyacrylamide gel (29 : 1 acrylamide-bisacrylamide), separated by SDS-PAGE, and transferred to PVDF membranes (Millipore, USA). The membrane was blocked for 1 h at room temperature in TBST buffer (50 mM pH 7.4 Tris-HCl, 150 mM NaCl, and 0.1% Tween-20) containing 5% nonfat dried-milk. The membrane was incubated with the appropriate primary antibody in TBST with 5% nonfat dried milk overnight at 4°C. Primary antibody anti-PGC-1*α* rabbit polyclonal antibody (SC-13067) (Santa Cruz, USA) was used to detect PGC-1*α*. Immunoblots were hybridized with antibody raised against GAPDH as loading control (Santa Cruz, USA). The membrane was then washed 3 times with TBST and incubated for 1 h with HRP-conjugated secondary antibody. Finally, the immunoreactive proteins were visualized using Chemiluminescence reagent (Amersham Biosciences, USA).

### 2.5. Statistical Analysis

Values are expressed as means ± SE. Statistically significant differences were determined using unpaired Student's *t*-tests. Statistical differences were considered significant if *P* < 0.05.

## 3. Results

### 3.1. Effect of Electroacupuncture on the Expression of PGC-1*α* and UCP-1 in the BAT of Normal Rats

**Figure 2:**
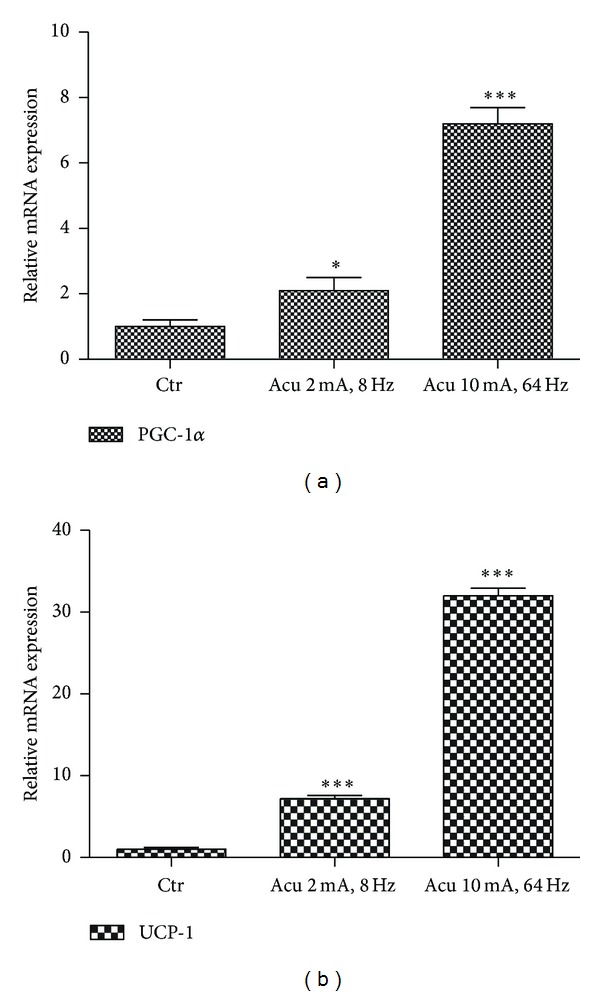
Effect of electroacupuncture on the expression of PGC-1*α* and UCP-1 in the BAT of normal rats. (a) Real-time PCR analysis of induction of PGC-1*α* mRNA in BAT of normal rats under different electroacupuncture conditions at ST36 acupoint. Relative expression was normalized to the expression of cyclophilin. Data represent mean ± SEM of at least three independent experiments. (b) Real-time PCR analysis of induction of UCP-1 mRNA in BAT of normal rats under different electroacupuncture conditions at ST36 acupoint.

PGC-1*α* is a crucial transcriptional coactivator for mitochondrial biogenesis and fatty acid oxidation. To investigate the effect of electroacupuncture on the gene expression of PGC-1*α* in brown adipose tissue (BAT), normal rats were accepted to be treated electroacupuncture at acupoint Zusanli (ST36) for three days at different stimulating conditions as described in Section 2. Our results indicated that electroacupuncture at ST36 acupoint significantly increased the expression of PGC-1*α* mRNA in the BAT of rats (Figure 2(a)). PGC-1*α* mRNAs were increased by 2-fold at EA (2 mA, 8 Hz) and 7-fold at electroacupuncture (10 mA, 64 Hz), respectively (Figure 2(a)).

PGC-1*α* coactivates the expression of UCP-1 which is linked to energy expenditure through uncoupled oxidative phosphorylation. Our data illustrated that the expression of UCP-1 mRNA in BAT was also induced by electroacupuncture at ST36 acupoint. UCP-1 mRNAs were increased by 8-fold at electroacupuncture (2 mA, 8 Hz) and 32-fold at electroacupuncture (10 mA, 64 Hz), respectively (Figure 2(b)). Although the expression levels of PGC-1*α* and UCP-1 mRNA are proportional to the intensity of EA stimulation (Figures 2(a) and 2(b)), the low intensity (2 mA, 8 Hz) was selected as experimental condition to sustain rats in a comfortable state.

### 3.2. Effect of Electroacupuncture on the Expression of PGC-1*α* and UCP-1 in the BAT of Obese Rats

**Figure 3:**
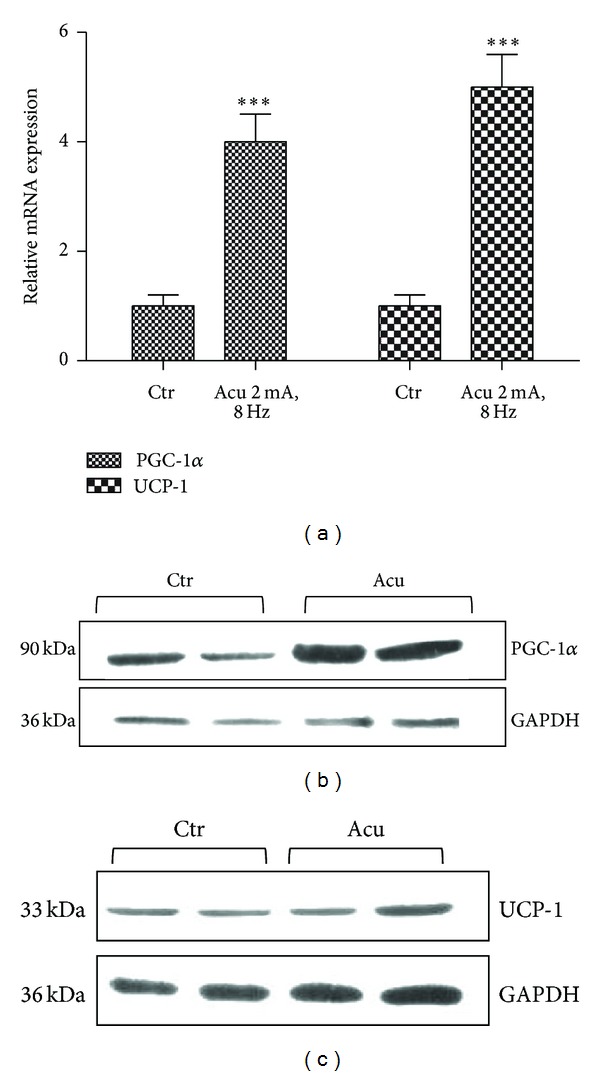
Effect of electroacupuncture on the expression of PGC-1*α* and UCP-1 in the BAT of obese rats. (a) Real-time PCR analysis of induction of PGC-1*α* and UCP-1 mRNA in the BAT of obese rats under low intensity electroacupuncture at ST36 acupoint. Relative expression was normalized to expression of cyclophilin. Data represent mean ± SEM of at least three independent experiments. (b) Western blot analysis of PGC-1*α* proteins expression in the BAT of obese rats after low intensity electroacupuncture treatment. GAPDH was blotted as a loading control. (c) Western blot analysis of UCP-1 proteins expression in the BAT of obese rats after low intensity electroacupuncture treatment. GAPDH was blotted as a loading control.

To further investigate the effect of electroacupuncture at acupoint ST36 on the expression of PGC-1*α* in the BAT of obese rats, obese rats were treated with electroacupuncture (2 mA, 8 Hz) at acupoint ST36. Electroacupuncture increased the expression of PGC-1*α* mRNA by 4-fold and UCP-1 mRNA by 5-fold in the BAT of obese rats, respectively (Figure 3(a)). And western bloting analysis showed that PGC-1*α* protein was significantly increased (Figure 3(b)), while UCP-1 protein was comparably induced in the BAT of obese rats by electroacupuncture at acupoint ST36 (Figure 3(c)).

### 3.3. Effect of Electroacupuncture on Gene Expression of Mitochondrial Respiratory Components in BAT

**Figure 4:**
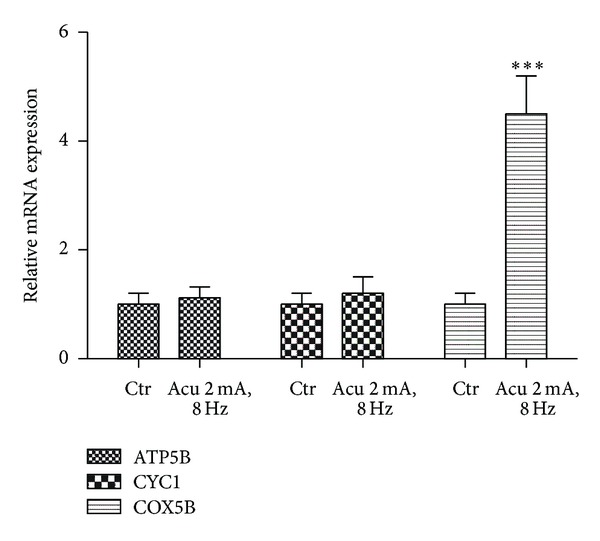
Effect of electroacupuncture on gene expression of mitochondrial respiratory complexes in BAT. Real-time PCR analysis of the expression of ATP5B, CYC1, and COX5B mRNA by electroacupuncture in the BAT of rats. Relative expression was normalized to expression of cyclophilin. Data represent mean ± SEM of at least three independent experiments.

To further study the effect of electroacupuncture on the expression of PGC-1*α* associated genes, the gene expressions of subunit B of ATP synthase (ATP5B), cytochrome c 1 (CYC1), and cytochrome c oxidase subunit Vb (COX5B) were analyzed which have been shown to be directly regulated by PGC-1*α* [19]. The expression of ATP5B and CYC1 mRNA were comparable between control and electroacupuncture group, but COX5B was increased significantly after electroacupuncture treatment (Figure 4). These nuclear-encoded proteins play an essential role in the regulation and assembly of the mitochondrial respiratory complexes.

### 3.4. Effect of Electroacupuncture on PGC-1*α* Expression in Skeletal Muscle of Rats

**Figure 5:**
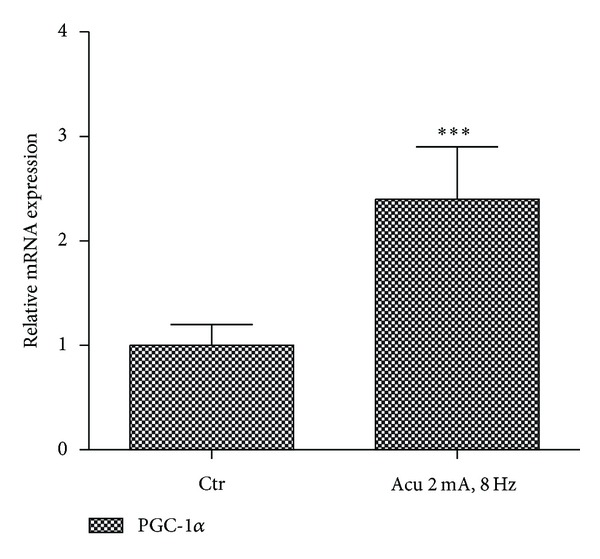
Effect of electroacupuncture on the expression of PGC-1*α* mRNA in skeletal muscle. Real-time PCR analysis of PGC-1*α* mRNA expression in skeletal muscle around acupoint Zusanli (ST36). Relative expression was normalized to expression of cyclophilin. Data represent mean ± SEM of at least three independent experiments.

Electroacupuncture stimulation will induce muscle contraction like exercise which has been observed to activate PGC-1*α* expression in muscle [20]. We measured the expression of PGC-1*α* mRNA in skeletal muscle around acupoint Zusanli (ST36) located in the hind leg of rats. The data illustrated that electroacupuncture induced the expression of PGC-1*α* mRNA in skeletal muscle by 2.5-fold (Figure 5). This result is similar to previous report that electric pulse stimulated PGC-1*α* expression in cultured murine muscle cells [21]. Electroacupuncture induced PGC-1*α* expression not only in rat BAT but also in rat skeletal muscle around acupoint ST36.

### 3.5. Effect of Electroacupuncture on Food Intake and Body Mass of Obese Rats

**Figure 6:**
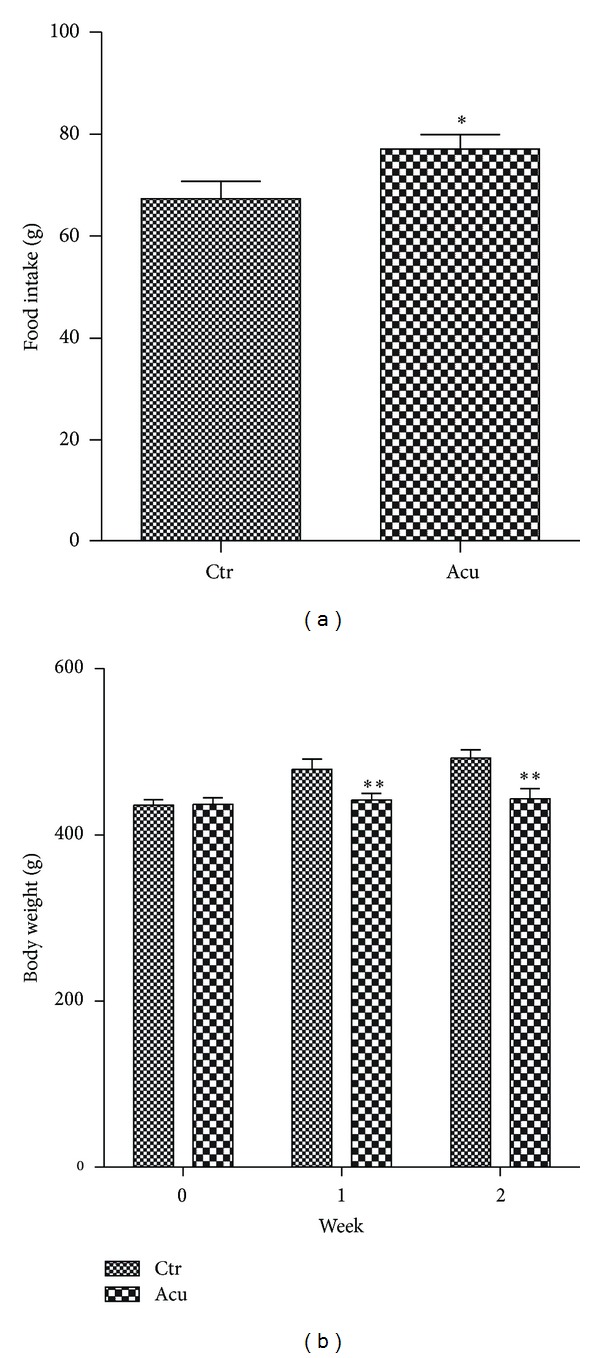
Effect of electroacupuncture on food intake and body weight of obese rats. (a) The total food intake of rats was statistically analyzed during the whole acupuncture treatment. Values are means ± S.E. (*n* = 6). (b) The body weight of rats was measured per week during electroacupuncture. Values are means ± S.E. (*n* = 6).

We measured food intake every day and body weight every week during electroacupuncture treatment. We found that electroacupuncture increased the total food intake of obese rats during the treatment period (Figure 6(a)). While, the body weight of obese rats in the electroacupuncture group was less than about 10% of control group after two weeks treatment (Figure 6(b)). We presumed that electroacupuncture reduced body weight of obese rats may be, or at least partially, through induction of the expression of PGC-1*α* and UCP-1 to increase the energy expenditure in BAT.

## 4. Discussion

Acupuncture is one of the oldest therapeutic interventions and is now widely accepted in the world. Acupuncture is considered to be effective and safe alternative medicine. To mimick the manual operation for the treatment of human diseases, rats were gently fixed by homemade holder and not anesthetized during electroacupuncture in this study (Figure 1(a)). In our early study that referred to previous experimental methods [22, 23], rats underwent electroacupuncture operation under anesthesia condition. We found that anesthesia method is not suitable to investigate the effect of electroacupuncture on the expression of PGC-1*α* in the BAT of rats, because anesthesis condition made rats in cold state which has been reported to highly induce PGC-1*α* expression in the BAT of mice and rats [14]. Electroacupuncture is a type of acupuncture wherein needles are attached to an apparatus that produces continuous electric pulses. The electroacupuncture parameters can be precisely controlled so the results are reproducible, whereas the outcome from manual acupuncture is operator dependent and therefore is not as reproducible. The intensity and frequency of electroacupuncture are easily adjusted by operator. Electroacupuncture rather than manual acupuncture has been used in the improvement of insulin sensitivity through activation of SIRT1/PGC-1*α* [24]. Low and high intensity electroacupuncture were performed in our study. Although high intensity electroacupuncture (10 mA, 64 Hz) induced higher expression of PGC-1*α*, low intensity electroacupuncture (2 mA, 8 Hz) was selected for this study to match manual acupuncture operation and maintain rats in a conscious and comfortable states in which rats is under quiet and happy state without anxiety and pain during whole experiments.

It is interesting that electroacupuncture at acupoint Zusanli (ST36) increased the expression of PGC-1*α* in the BAT of rats. It has been reported that electroacupuncture (EA) can promote expression of uncoupling protein-1 (UCP-1) and *β*_3_-adrenoceptor (AR) in BAT [25]. *β*_3_-adrenergic receptor (AR) is expressed abundantly and predominantly in BAT and plays an important role in the modulation of this uncoupling oxidative phosphorylation process. Catecholamines are endogenous agonists against *β*_3_-adrenergic receptor (AR). PGC-1*α* coactivates UCP-1 expression to link *β*_3_-adrenergic receptor activation to adaptive thermogenesis in BAT [15]. Induction of PGC-1*α* by cold exposure is largely due to sympathetic nervous system input through *β*-adrenergic receptors. It has been reported that electroacupuncture at Zusanli (ST36) increased the contents of dopamine and serotonin and acupuncture enhances the synaptic dopamine availability to improve motor function in a mouse model of Parkinson's disease [26, 27]. Therefore, we presumed that the expression of PGC-1*α* in the BAT of rats by EA at Zusanli (ST36) might be activated through sympathetic nervous system (SNS) and catecholamine action. To confirm the conclusion, we should conduct a series of further controls in the future experiments, such as the acupuncture without any electrical stimulation and acupuncture at a different site. In this study, we mainly focused on whether the expression of PGC-1*α* in BAT could be induced in BAT by electroacupuncture at Zusanli (ST36). A large number of selective agonists of *β*_3_-adrenergic receptor have been synthesized; however, none was finally commercialized because of the short-time span of their efficacy and the rapid downregulation of the receptors [28, 29]. Our results indicated that electroacupuncture at acupoint ST36 is a safe and effective alternative approach to induce PGC-1*α* expression in BAT.

## Figures and Tables

**Table 1:** Primer sequences for PCR analysis.

Gene	Forward primer	Reverse primer
PGC-1*α*	5′-TGC CAT TGT TAA GAC CGA G-3′	5′-GGT CAT TTG GTG ACT CTG G-3′
UCP-1	5′-GAT CCA AGG TGA AGG CCA GG-3′	5′-GTT GAC AAG CTT TCT GTG GTG G
ATP5B	5′-GGC ACT GAA GGC TTG GTT AG-3′	5′-CAA GAG AAG ATT CTC AGC GAC-3′
CYC1	5′-CCC TGA CCT CAG CTA CAT C-3′	5′-CAA GAG AAG ATT CTC AGC GAC-3′
COX5B	5′-GCT GCA TCT GTG AAG AGG ACA AC-3′	5′-CAG CTT GTA ATG GGT TCC ACA GT-3′
Cyclophilin	5′-CCA TCG TGT CAT CAA GGA CTT CAT-3′	5′-CTT GCC ATC CAG CCA GGA GGT CTT-3′

PGC-1*α*: peroxisome proliferators-activated receptor-*γ* coactivator-1*α*; UCP-1: uncoupling protein-1; ATP5B: ATP synthase B subunit; CYC1: cytochrome C unit 1; COX5B: cytochrome oxidase 5 B subunit; cyclophilin: peptidylprolyl isomerase.

## References

[1] Faeh D, Braun J, Tarnutzer S, Bopp M (2011). Obesity but not overweight is associated with increased mortality risk. *European Journal of Epidemiology*.

[2] Katzmarzyk P, Reeder B, Elliott S (2012). Body mass index and risk of cardiovascular disease, cancer and all-cause mortality. *Canadian Journal of Public Health*.

[3] Halford J (2006). Obesity drugs in clinical development. *Current Opinion in Investigational Drugs*.

[4] Belivani M, Dimitroula C, Katsiki N (2013). Acupuncture in the treatment of obesity: a narrative review of the literature. *Acupuncture in Medicine*.

[5] Sui Y, Zhao HL, Wong VC (2012). A systematic review on use of Chinese medicine and acupuncture for treatment of obesity. *Obesity Reviews*.

[6] Cho S-H, Lee J-S, Thabane L, Lee J (2009). Acupuncture for obesity: a systematic review and meta-analysis. *International Journal of Obesity*.

[7] Cypess AM, Lehman S, Williams G (2009). Identification and importance of brown adipose tissue in adult humans. *The New England Journal of Medicine*.

[8] van Marken Lichtenbelt WD, Vanhommerig JW, Smulders NM (2009). Cold-activated brown adipose tissue in healthy men. *The New England Journal of Medicine*.

[9] Virtanen K, Lidell M, Orava J (2009). Functional brown adipose tissue in healthy adults. *The New England Journal of Medicine*.

[10] Zingaretti M, Crosta F, Vitali A (2009). The presence of UCP1 demonstrates that metabolically active adipose tissue in the neck of adult humans truly represents brown adipose tissue. *The FASEB Journal*.

[11] Ouellet V, Labbé SM, Blondin D (2012). Brown adipose tissue oxidative metabolism contributes to energy expenditure during acute cold exposure in humans. *Journal of Clinical Investigation*.

[12] van der Lans A, Hoeks J, Brans B (2013). Cold acclimation recruits human brown fat and increases nonshivering thermogenesis. *Journal of Clinical Investigation*.

[13] Bartelt A, Bruns OT, Reimer R (2011). Brown adipose tissue activity controls triglyceride clearance. *Nature Medicine*.

[14] Puigserver P, Wu Z, Park C, Graves R, Wright M, Spiegelman BM (1998). A cold-inducible coactivator of nuclear receptors linked to adaptive thermogenesis. *Cell*.

[15] Puigserver P, Spiegelman BM (2003). Peroxisome proliferator-activated receptor-*γ* coactivator 1*α* (PGC-1*α*): transcriptional coactivator and metabolic regulator. *Endocrine Reviews*.

[16] Lin JD, Handschin C, Spiegelman BM (2005). Metabolic control through the PGC-1 family of transcription coactivators. *Cell Metabolism*.

[17] Collins S, Yehuda-Shnaidman E, Wang H (2010). Positive and negative control of Ucp1 gene transcription and the role of *Β*-adrenergic signaling networks. *International Journal of Obesity*.

[18] Zhang Y, Huypens P, Adamson AW (2009). Alternative mRNA splicing produces a novel biologically active short isoform of PGC-1*α*. *Journal of Biological Chemistry*.

[19] Choi C, Befroy D, Codella R (2008). Paradoxical effects of increased expression of PGC-1*α* on muscle mitochondrial function and insulin-stimulated muscle glucose metabolism. *Proceedings of the National Academy of Sciences of the United States of America*.

[20] Miura S, Kawanaka K, Kai Y (2007). An increase in murine skeletal muscle peroxisome proliferator-activated receptor-*γ* coactivator-1*α* (PGC-1*α*) mRNA in response to exercise is mediated by *β*-adrenergic receptor activation. *Endocrinology*.

[21] Burch N, Arnold A, Item F (2010). Electric pulse stimulation of cultured murine muscle cells reproduces gene expression changes of trained mouse muscle. *PLoS ONE*.

[22] Mannerås L, Jonsdottir IH, Holmäng A, Lönn M, Stener-Victorin E (2008). Low-frequency electro-acupuncture and physical exercise improve metabolic disturbances and modulate gene expression in adipose tissue in rats with dihydrotestosterone-induced polycystic ovary syndrome. *Endocrinology*.

[23] Onda A, Jiao Q, Nagano Y (2011). Acupuncture ameliorated skeletal muscle atrophy induced by hindlimb suspension in mice. *Biochemical and Biophysical Research Communications*.

[24] Liang F, Chen R, Nakagawa A (2011). Low-frequency electroacupuncture improves insulin sensitivity in obese diabetic mice through activation of SIRT1/PGC-1*α* in skeletal muscle. *Evidence-Based Complementary and Alternative Medicine*.

[25] Liu Z, Sun F, Zhao D (2003). Effect of acupuncture on uncoupling protein 1 gene expression for brown adipose tissue of obese rats. *Chinese Journal of Integrative Medicine*.

[26] Kim S, Doo A, Park J (2011). Acupuncture enhances the synaptic dopamine availability to improve motor function in a mouse model of Parkinson’s disease. *PLoS ONE*.

[27] Shou Y, Yang Y, Xu M (2013). Electroacupuncture inhibition of hyperalgesia in rats with adjuvant arthritis: involvement of cannabinoid receptor 1 and dopamine receptor subtypes in striatum. *Evidence-Based Complementary and Alternative Medicine*.

[28] Whittle AJ, López M, Vidal-Puig A (2011). Using brown adipose tissue to treat obesity—the central issue. *Trends in Molecular Medicine*.

[29] Hirschberg V, Fromme T, Klingenspor M (2011). Test systems to study the structure and function of uncoupling protein 1: a critical overview. *Frontiers in Endocrinology*.

